# Staying safe, feeling welcome, being seen: How spatio‐temporal configurations affect relations of care at an inclusive health and wellness centre

**DOI:** 10.1111/hex.13858

**Published:** 2023-09-01

**Authors:** Stefanie Plage, Kirsten Baker, Cameron Parsell, Rose‐Marie Stambe, Ella Kuskoff, Arif Mansuri

**Affiliations:** ^1^ ARC Centre of Excellence for Children and Families over the Life Course Indooroopilly Australia; ^2^ Australian Research Centre in Complementary and Integrative Medicine The University of Technology Sydney Broadway Australia; ^3^ Inclusive Health and Wellness Hub South Brisbane Australia

**Keywords:** Australia, housing instability, primary health care, qualitative research, relations of care, service delivery

## Abstract

**Background:**

People experiencing homelessness also experience poorer health and frequently attend acute care settings when primary health care would be better equipped to meet their needs. Existing scholarship identifies a complex mix of individual and structural‐level factors affecting primary health care engagement driving this pattern of health services utilisation. We build on this existing knowledge, by bringing the spatio‐temporal configurations of primary health care into focus. Specifically, we interrogate how space and time inflect situated practices and relations of care.

**Methods:**

This study took an ethnographic approach and was conducted 2021–2022 at an inclusive health and wellness centre ("the Centre") in Southeast Queensland, Australia. The data consists of 46 interviews with 48 people with lived experience of homelessness, including participants who use the services offered at the Centre (*n* = 26) and participants who do not (*n* = 19). We also interviewed 20 clinical and non‐clinical service providers affiliated with the Centre and observed how service delivery took place. Interviews and observations were complemented by visual data, including participant‐produced photography. All data were analysed employing a narrative framework.

**Results:**

We present three interrelated themes demonstrating how space and time affect care, that is ‘staying safe’, ‘feeling welcome’ and ‘being seen’. ‘Staying safe’ captures the perceptions and practices around safety, which sit in tension with making service users feel welcome. ‘Feeling welcome’ attends to the sense of being invited to use services free of judgment. ‘Being seen’ depicts capacities to see a health care provider as well as being understood in one's lived experience.

**Conclusion:**

Spatio‐temporal configurations, such as attendance policies, consultation modalities and time allocated to care encounters afford differential opportunities to nurture reciprocal relations. We conclude that flexible service configurations can leverage a relational model of care.

**Patient or Public Contribution:**

Service providers were consulted during the design stage of the project and had opportunities to inform data collection instruments. Two service providers contributed to the manuscript as co‐authors. People with lived experience of homelessness who use the services at the inclusive health centre contributed as research participants and provided input into the dissemination of findings. The photography they produced has been featured in an in‐person exhibition, to which some have contributed as consultants or curators. It is hoped that their insights into experiences of welcomeness, safety and being seen will inform flexible and relational primary health care design, delivery, and evaluation to better cater for people experiencing housing instability and poverty.

## 1 INTRODUCTION

Primary health care is the cornerstone of health care in Australia^1^ and other OECD (i.e., Organization for Economic Cooperation and Development) countries like the United Kingdom^2^ and United States.^3^ However, people with experiences of homelessness internationally report receiving inadequate primary health care characterised by judgement and discrimination.^4^, ^5^ This compounds poor health among this cohort known to have high levels of chronic disease, mental ill‐health, and comorbidities.^6^ The OECD^7^ reports that people who are homeless die on average 30 years earlier than the non‐homeless population. Acute care settings are ill‐equipped to deliver complex health and social care, yet, people who are homeless appear to visit emergency departments more frequently than non‐disadvantaged peers^8^, ^9^, ^10^, ^11^ (see also^12^). This pattern of health services utilisation is explained by a mix of individual and structural‐level factors affecting primary health care engagement^13^ which often centre the health‐related behaviours of the individuals who seek care.^14^ We expand this focus to interrogate the configurations of service delivery through which primary health care is provided to people who are homeless. Findings are relevant to policy makers and health practitioners given the present rigidity of the funding structure for primary health service provision, where standard long appointments and high non‐attendance rates are a potential source of pressure for general practice and its financial viability.

Our study is underpinned by the understanding that health care comprises a set of practices that are embedded within broader structures and relations.^15^, ^16^ Social actors, organisations, and cultural context bear upon the collective practices through which a caring relation is enacted. Specifically, relations of care are embedded within spatial and temporal parameters which inflect what can(not) be done. In response to systemic failures, emergent models of primary health care sitting on the margins of health systems seek to increase inclusion of people who are homeless in relations of care.^17^, ^18^ Drawing on ethnographic research at such an inclusive health and wellness centre, we examine how spatial and temporal configurations afford opportunities to establish and nurture caring relations.

### 1.1 Background

Australia, where this research took place, features Medicare, a tax‐funded and compulsory health insurance scheme that is accessible to citizens and permanent residents augmented by voluntary but incentivized private health insurance.^19^ Despite the existence of a universalist health care scheme, health disparities for homeless people persist.^8^, ^13^, ^20^ A person is considered homeless for the purpose of the Australian Census^21^ ^,p.19^ if they are either, ‘entirely roofless’ or occupy a dwelling that ‘is physically inadequate, provides no tenure, or only a short and non‐extendable tenure, or enables the resident no control of, and access to, space for social relations’. This definition of homelessness accommodates profoundly different housing arrangements and opportunities to engage with primary health care.^16^

Conceptualising primary health care as delivered *within* social relations, throws into relief how spatial and temporal practice standards, such as appointment length, modalities in delivery, attendance, and behavioural policies promote or hinder greater health equity. A lack of consultation time has been reported by general practitioners (GPs) as a barrier to developing trusted relationships with patients.^22^ Luchenski et al.,^23^ observed that taking time for appointments to allow for listening to patients is important for inclusive healthcare. A qualitative review of the literature on health seeking experiences amongst marginalised groups in urban Australia noted how a lack of clear communication from healthcare providers risked confusion for patients when time constraints affect the care that can be provided.^4^ Appointment lengths of 25–30 min are considered best practice for people with experience of homelessness, who disproportionally present as tri‐morbid (i.e., the co‐presence of a mental health condition, substance use, and chronic disease).^13^ However, health system logics incentivize short appointment durations with repercussions for the financial viability of general practices.

People experiencing homelessness may find attending scheduled appointments difficult due to transportation issues, keeping track of and prioritizing commitments, or competing pressures (i.e., securing belongings, food, or shelter).^14^, ^24^ Punitive ramifications for missing scheduled appointments (e.g., exclusion from services) are a widely accepted practice.^4^ Working punitively fails to recognise homeless people's agency,^25^ undermines health seeking, impacts therapeutic relationships and further entrenches marginalisation when greater levels of support are needed.^26^ Wen et al.^27^ found that perceptions of being unwelcome affected the disposition to seek health care; while welcoming, friendly environments with flexible appointment structures have been shown to improve access to primary health care for socially disadvantaged populations.^28^ Drop‐in models and after‐hours services, incorporating peer navigators, appointment reminders and the integration of primary and behavioural care diversified to include allied and complementary therapies, have been proposed to make primary health care more accessible.^4^, ^5^

We contribute to this scholarship by exploring how spatio‐temporal configurations of an inclusive primary health care centre are leveraged to enable a relational model of care. Taking a relational approach to the analysis, we report on three interrelated themes—feeling welcome, staying safe and being seen, and conclude by considering implications for inclusive health practice and policy.

## 2 MATERIALS AND METHODS

### 2.1 The setting

The present study was undertaken at an inclusive health centre (‘the Centre’), a not‐for‐profit health service providing care at multiple sites across an urban location in Southeast Queensland, Australia. The Centre was established to overcome health inequality and is a resource within the community for people experiencing one or more of the following: homelessness, disability, poverty, mental illness, chronic disease and/or substance use. The core services are co‐located and include GP and nursing, as well as wellness services (i.e., acupuncture, shiatsu, myotherapy). These are complemented regularly by specialist services, such as podiatry, dental, HEP C, vaccination, and endocrine clinics. For the 2020/2021 financial year 3933 GP and 374 nurse practitioner consultations were held at the Centre, complemented by 1320 acupuncture and 875 other (e.g., massage, reflexology, podiatry) sessions. Outreach services and telehealth resulted in a significant number of additional nurse appointments (i.e., 2796). Massage, myotherapy and GP consultations take place in private rooms. By default, acupuncture is delivered in a communal space. However, service users can opt for greater privacy too. Early 2022, significant flood damage to the Centre's main campus impacted service delivery and data collection for this study. In response, the Centre opened pop‐up sites to minimize disruption of services. The resulting dispersion of core services provided opportunities to reflect on how spatio‐temporal configurations affect relations of care.

### 2.2 Study design

We took an ethnographic approach to data collection at the Centre, including observations of care encounters, participant‐produced photography, and interviews carried out between November 2021 and October 2022. The partner organisation facilitating fieldwork operates the Centre alongside other social care and outreach services (e.g., housing support), giving us access to both people using the Centre's services and those who do not (i.e., summarily defined here as ‘potential service users’). Notably, non‐service users are included in the analysis to discern nuances in health care experiences; these contrasts stand out especially in the theme ‘Being Seen’.

To be eligible for study participation, potential service users needed to fit at least one of three criteria: (i) being marginally housed; (ii) having sought homelessness services for the first time in the 12 months before the study; or (iii) falling within the definition of chronic homelessness, with at least one debilitating health condition and multiple episodes of homelessness.^29^ After ethical approval (2021/HE002663) by the first author's institutional Human Research Ethics Committee, we employed a theoretical sampling strategy and initially invited clinical and non‐clinical, volunteer, and salaried Centre staff and partners, and later potential service users in its catchment to participate in the study. Staff were approached via an invitation email to capture the breadth of service delivery experiences at the Centre. Recruitment continued until we were satisfied that all relevant service offerings and modalities were included. To recruit potential service users, the partner organisation assessed if it was appropriate to approach a person regarding study participation and facilitated the introduction to a member of the research team. Where consent was forthcoming, a mutually convenient time for an in‐person interview was arranged.

Two separate interview guides were developed for the participant groups. Topics covered domains of primary health care, such as continuity of care, health promotion, preventative health, holistic medicine, and self‐care. Interviewing unfolded iteratively, with insights gained from early interviews used to refine the questions and probes in later ones. The spatial and temporal configurations of service delivery were identified early in the process as an analytical focus to understand experiences with health care. Participants with experience of homelessness were invited after the initial interview to participate in a photography component of the study. Consenting participants were given a digital camera with a photo assignment asking them to ‘tell the story of what health looks and feels like and what it means to them’. The photographic activity was discussed, and photographs captioned during a final photo‐elicitation interview (PEI). The first author also took photographs during data collection where permitted.

### 2.3 Data analysis

Observation notes were typed up. Interviews were audiorecorded, professionally transcribed and deidentified. One service provider and one non‐service user wished not to be recorded and interview notes were summarised for analysis. Data were analysed supported by NVivo12 software combining four types of narrative analysis ‐ thematic, structural, dialogic, and visual^30^ to integrate insights from observations, interviews, and photographs and from different study participants. This complies with principles of rigour in qualitative research.^31^ Thematic analysis is focussed on a narrative's content; structural analysis explores the forms of storytelling (e.g., metaphors); dialogic analysis acknowledges that narratives are co‐produced, and visual analysis examines visual content alongside text.^30^ Text‐based, observational, and visual data were given equal importance in the analysis. Photographs were inserted into transcripts before coding. After reading and rereading all transcripts and notes, annotation and cross‐comparison, codes were aggregated into themes. Frequent team dialogues served to add depth to themes and reflect on their complexity against what we observed in practice. During these discussions, the cultivation of reciprocal relations was identified as a key process in making sense of themes. The analysis was completed by applying this conceptual lens upon revisiting the coded data, situating findings within the extant literature, and seeking feedback from stakeholders at the Centre.

## 3 RESULTS

We report findings from 46 interviews with 48 potential service users (30 male, 16 female, one trans male and one gender diverse) and 20 service providers (four non‐clinical, four GPs, six nurses, one pharmacist, five wellness) working internally (17) or partnering (three) with the Centre. Interviews with service providers lasted 26–74 min (average 44 min), and interviews with homeless participants lasted 10–105 min (average 45 min). Among the latter, 19 had reportedly not used the Centre's services; 26 had engaged with the Centre's GP and/or wellness offerings. Interview data was complemented through 65 h of observations of clinical and non‐clinical care encounters across four different Centre sites, and 15 h of observations during outreach activities. Fourteen participants completed the PEI lasting between 42 and 222 min (average 101 min), contributing overall 680 unique photographs for analysis. We report how spatio‐temporal configurations at the Centre are leveraged to enable a relational model of care in which people with experience of homelessness are made to feel safe, welcome, and seen. All names are pseudonyms.

### 3.1 Staying safe

No incidences of escalation of conflict into interpersonal violence during care encounters were observed during the time spent at the Centre and none of the interviewed service providers reported experiences of victimization when probed. Nonetheless, the potential of violence was conspicuously addressed at the Centre in the organisation of space, behaviour management strategies and targeted training, as well as technologies, such as duress buttons and security on call. This service provider admitted:

Some of the behaviours of patients can be challenging at times. There have been times where I've been threatened, there's times when I've felt scared. And certainly, that has improved with time. … If they're continuing to come back, I don't feel threatened by these patients. [Brent, service provider]

Brent's acknowledgement of challenging behaviours is followed by what he considered the greatest mitigating factor to these challenges: an ongoing relationship with a patient. This resonates with the experiences many clinical and nonclinical staff and volunteers shared, in which building trust was identified as the basis for a caring relationship in which the potential for violence was backgrounded. Notably, the concern with safety was as much about staff staying safe at work, as it was about making service users, who had often experienced trauma, feel safe. Many service providers explained how they made the configuration of space work for them:

I'm super conscious around how I engage people. I move my chair, I do whatever I need to do. … The other day, I had a table here [points away from her] … It's just too much of a distance. … I want them to feel safe, I want them to feel respected, and I want to build trust, and I want them to know who I am. … I don't really feel unsafe. [Cathy, service provider]

Many service providers, like Cathy, worked in outreach and pop‐up clinical settings meeting service users at their usual whereabouts. For service users this was not only convenient but also helped them to feel secure in their surroundings. We often heard narrations of how service providers arranged furniture or adjusted body posture and position to reduce physical distance and appear approachable, often without the benefit of a purpose‐built environment, such as a clinical office. As the impact of the floods on the Centre lingered, similar strategies were also employed in the temporary locations where primary care and wellness services relocated.

**Figure 1:**
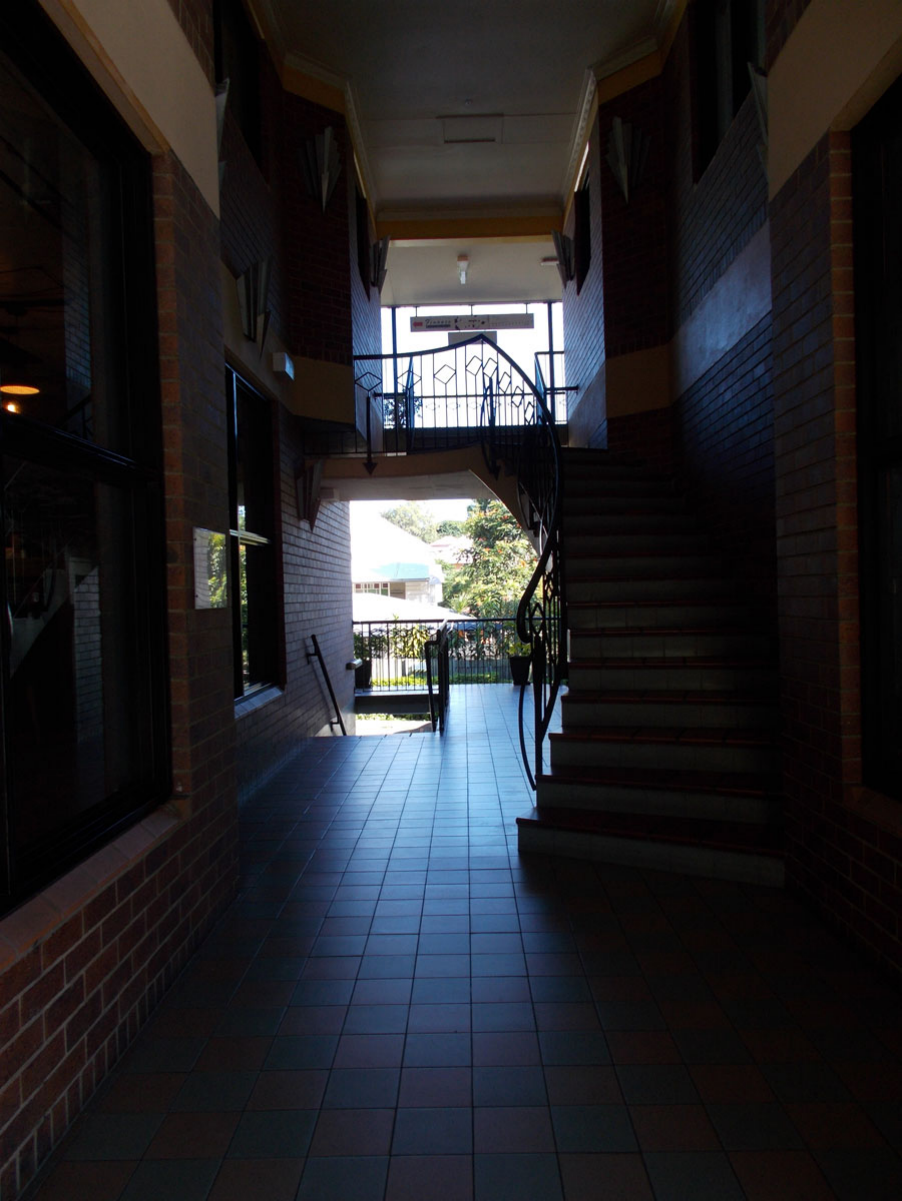
Maddie: ‘when we've got safe people, it makes the place safe’.

Wellness service user, Maddie, for example, photographed the dark staircase which leads to the pop‐up wellness service she attended (see Figure 1).

While lamenting the loss of the previous wellness space with its bespoke and inviting ambience, comfortable chairs and heat lamps, Maddie's main concern was with safety. She commented on the photograph:

when we've got safe people, it makes the place safe [Maddie, service user]

Safety was tied up with being able to trust the Centre staff and volunteers to recognise and respond to immediate service user needs. This was evident in the practices during wellness treatment as the following observation shows:

Maddie arrives and after being welcomed by the wellness coordinator takes a seat next to me to wait her turn. We chat animatedly and Maddie appears to be looking forward to the acupuncture. Her mood quickly changes after settling in the reclining chair. She is trembling and silently crying as the wellness coordinator comforts her by gently touching her hand. She continuously checks in with Maddie, speaking to her softly as she completes the treatment. [Observation notes, communal space]

Being able to complete a 20‐min treatment is a remarkable outcome for service users with a history of victimization. We observed hypervigilant or restless service users struggling with even a small wait for a consultation at the GP clinic. Many Centre staff and volunteers were observed to practice in line with trauma‐informed care models to support service users before, during and after consultation.^32^ Experiences like Maddie's indicate a tacit understanding between service providers and users leveraging nonverbal reassurance (i.e., touch) and ambience (i.e., soft lighting and instrumental music) to manage distress. Practices around safety extend into group settings such as open treatment spaces and waiting areas. Consider the following situation:

I had a man come in… I hadn't met him before, and he sort of walked into the community space and there were people in each of the chairs. … really straight up to me, and he was loud as well… It perhaps disrupted the sense of safety in the space momentarily. … we walked out of the hall together and down to the café. [Deirdre, service provider]

The same man returned another day to the communal space where the following was observed:

The wellness coordinator welcomed him and offered acupuncture, which he did not seem to be interested in. As the wellness coordinator listened to him talking noisily, she gently directed him away from the treatment area and the other service users until he left the premises towards the café. [Observation notes, communal space]

In this context, the importance of valued relationships between service users and providers often predating the dispersal of services came to light. Service users relied on the wellness coordinator to create a safe treatment space, and we observed similar strategies employed by non‐clinical staff in communal waiting areas. Note that while the potential of interpersonal conflict holds a presence, it is never taken as grounds to refuse treatment or permanently exclude a person from care at the Centre: the man was again offered treatment. Nonetheless, tensions were palpable between the themes of staying safe and making people feel welcome as we explore further in the next section.

### 3.2 Feeling welcome

Safety was an ongoing concern for service users. Service providers deliberately worked to make people attending their consultations feel safe, concurrently anticipating challenging behaviours from a minority of service users. This points to an important balance to be struck between behaviour management and making people feel welcome to attend service offerings. A non‐clinical service provider explained:

We'll put up with you not managing very well, being distressed, being upset, being angry, but there is a limit to what's acceptable. And generally it's at the point in which other patients are becoming involved or they're making direct threats … it is explained, ‘You won't be seeing that doctor again. You can go and see another doctor. But if you keep doing that, we will keep moving you around’. [Ally, service provider]

Service providers manning the reception saw their role as mitigating the immediate impact of challenging behaviours on other service users and providers.^33^ The above quote shows how behaviours were explained as driven by frustration. This allowed service providers’ to frame their responses as flexibility, rather than punishment. Instead of excluding individuals from service, they sought to match service users with service providers with whom a caring relationship could be cultivated. At times this prevented disengagement from primary healthcare, and at other times, it left a door open to reengage when desired. This approach to behaviour management implicitly acknowledges that health service delivery takes place within a reciprocal relationship. A service provider summarised:

everybody is always welcome … never turn somebody away … it feels like it's a space created for them, and encourage for them to come because they get benefit from the treatment [and] the rapport that they have. [Cara, service provider]

This was an oft‐mentioned service principle shared across all Centre staff and volunteers and echoed in the service user data. Some service users, like Maddie, compared how she felt at the Centre with previous experiences elsewhere:

I just feel welcome [at the Centre]. … my long‐term GP, whom I had a great deal of respect for, I broke down in the surgery. And he and his receptionist couldn't get me out fast enough. They just shoved me out the door. … My [previous] doctor didn't want anything to do with me after that. I tried to make an appointment. I was no longer welcome. [Maddie, service user]

Maddie felt let down by her mainstream GP in her hour of need, resulting in distrust and a breakdown of the caring relationship. In contrast, she ‘just felt welcome’ at the Centre and was supported through her distress. We also heard stories of how the Centre actively enabled primary health care engagement, beyond keeping an open‐door and providing emotional support, for example by arranging transport to and from appointments. Bill explained:

I felt good, stress‐free. … they took me to appointments, medical, to see the doctor and they drop you off. … just can't get to the places now that I've got ill health with emphysema, COPD. I wouldn't be able to cope with the hills and walking all that far. [Bill, service user]

**Figure 2:**
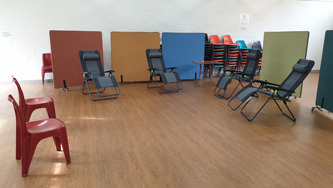
Taken by the first author with permission.

Transport arrangements were more than a practical aid to make sure service users do not miss scheduled appointments. Transport reinforced a sense of being welcome to benefit from Centre services on offer. The dispersion of services after flooding posed a serious challenge to the Centre's presence in the community. Timely resumption of services—albeit in a different location and with limitations—was supported by transport arrangements and enabled continuity in relations predating the flood event. Feeling welcome was also furthered by sharing ownership of Centre space. Consider the following observation together with the photograph of the set up (see Figure 2).

The door to the centre is open. Other people arrive before the wellness coordinator. One of them begins setting up the temporary treatment space: four reclining chairs in a semi‐circle and partitioning. I ask him if he works at the venue, and he denies that. When the wellness coordinator arrives, it becomes clear that he is a regular service user. [Observation notes, communal space]

These actions indicate the level of comfort felt in participating in services, as well as the affordances of being in an ad hoc setting. Allowing access to equipment and the location's facilities demonstrate the mutual relation of trust. We often observed service users arriving before the commencement of services, not only contributing to the spatial arrangement but settling in and conversing with one another. Likewise, service users waiting in the primary care pop‐up clinic frequently engaged in conversations with service providers and other service users while waiting to be seen. Figure 2 illustrates, how spatial setup might enhance a sense of reciprocity: chairs are arranged so that wellness service users face one another while treatments are provided. All treatments are done distally, minimizing the need to take off clothing. Somewhat surprisingly for us, privacy did not seem a concern among the wellness service users we interviewed, but private sessions could be accommodated within the flexible service delivery model, where desired. Indeed, idiosyncrasies in service delivery modalities across different practitioners supported a match‐making approach beyond the behaviour management strategies identified above (i.e., pairing service users with a suitable service provider within the Centre). The potential to be overlooked, let down and not having any options at all to meet health care needs could be mitigated in this way, as we elaborate below.

### 3.3 Being seen

Feelings of being safe, welcome, and able to see a doctor or health practitioner are afforded within the spatio‐temporal configurations in which care is done. The relational model of care here plays out in attendance policies which determine if a person is seen in primary health care, when and for how long. Notably, we found great variation in the practices at the Centre across its staff and volunteers:

I do flexible appointments. And some people are early birds, some people are afternoon people, some people are Saturday people. It's a conversational discussion and just knowing how people work and what's going to work and knowing their weekly routines. It's much more of a personal dynamic for me. I've got to know people quite well over the years [Eddie, service provider]

you're not going to be able to find them again. … [the Centre is] just more willing to work creatively like that, because you realise opportunistic is a real, big thing, of when you see that person, they need to be seen. … when you're working together closely like that, from the streets to the clinic, I think you've got that ability. Whereas if you're just with any GP, it's just a clean ‘no’, often. [Belinda, service provider]

A service user commented:

I would like the more structured approach because it's one of the things that I try to maintain in my own life, a little bit of structure and predictability. On the other hand, the positive effects of being able to access this service means I'm happy to take compromises. … if this means shifting booked appointments a little bit back, that's just fair enough. [Kevin, service user]

While the wellness space was operated as a drop‐in centre at the time of data collection, other wellness practitioners followed an appointment model, while the GP clinic ran a flexible booking system. This is an important finding, given the constraints coming from exclusively operating as *either* drop‐in *or* appointment‐based service. Concurrently, the service users we interviewed had differing preferences for how they arranged consultations. What was evident in those discussions across the data was once more the pivotal role of flexibility and the capacity to build enduring relationships of care in which different notions of what works could be accommodated. This was also apparent in considerations with respect to appointment length for GPs. This service provider revealed:

we wanted to give extra time, as mostly the histories are quite complex. You can't cover within 15 minutes as you see in other GP. … that extra bit of time, that creates that doctor‐patient relationship, therapeutic relationship. It helps that somebody's listening to them. [Basr, service provider]

This was mirrored in the experiences of service users we interviewed:

[GP] would take the time to understand our needs, apart from the medical, and what's happening in our life. So, he has an overview of what happens that would impact our physical and mental health. I think he looks after both the physical and the mental. And I've been seeing [GP] for a few years. [Sue, service user]

I had to change my doctor because he wasn't very nice to me. He wouldn't let me finish, he wouldn't listen, he was talking over the top of me … [Centre GP] wants the best for me and, yeah, she doesn't tell me what to do. And if she says no, she explains why. So, it's all good. And she listens and she feels and understands what I go through. [Anita, service user]

[GP] doesn't miss one trick, which is good. And she's slowly getting things sorted out for me…. it was a couple of days after I'd been here [at crisis accommodation]. … I was expecting [GP consultation] just to be like a catch‐up, and I was in there for just over an hour. [Heath, service user]

**Figure 3:**
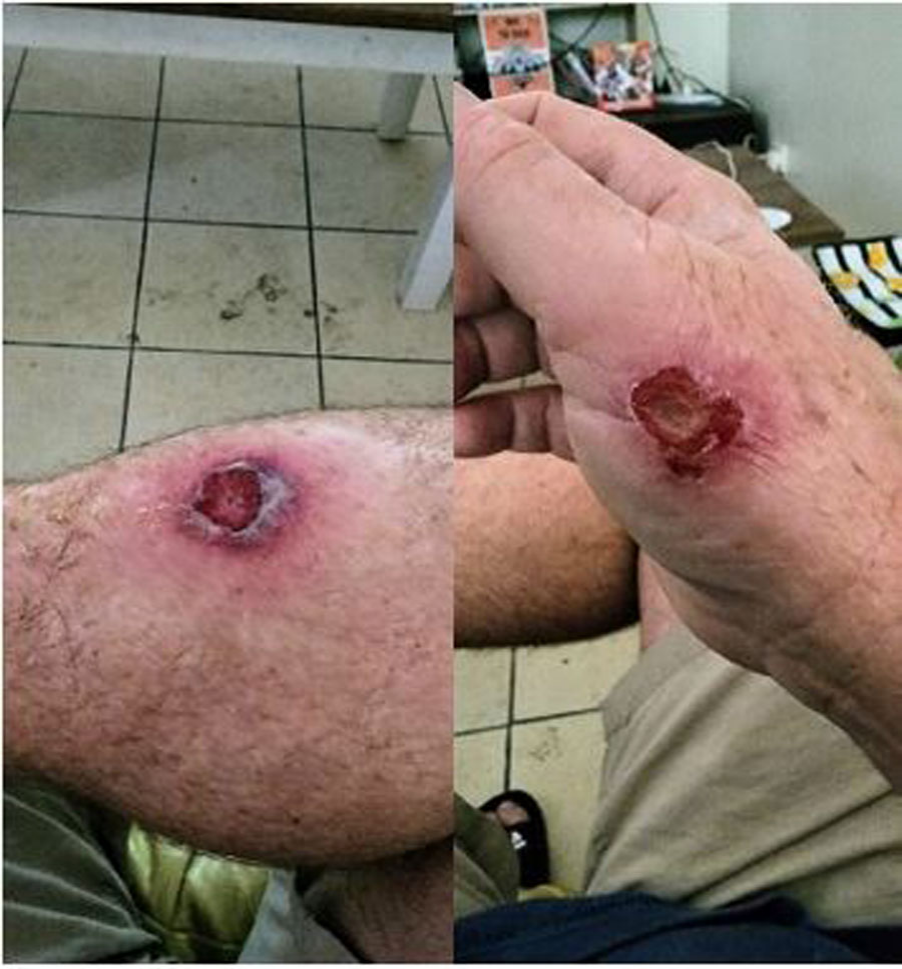
Nate: ‘when I get a chance, I've got to get the antibiotics going’.

Contrast these stories with non‐service user Nate's experience in seeking a consultation in mainstream primary health care. Nate lost income due to injuries for which he presented to an acute care service. There he was encouraged to seek out a GP for wound care (see Figure 3).

At the mainstream primary health care practice, he was asked to pay an out‐of‐pocket contribution, which he could not afford:

Saturday was the absolute kick in the teeth for me. I'm like, ‘Well, why bother?’ I would rather sit at the hospital for 12 hours, possibly never getting seen. … it makes me real shady about even trying to rebook a follow‐up [GP] appointment… [just] go back up to the ED even though the GPs open, because I'm really not trusting of the GP. [Nate, non‐service user]

Instead, Nate sought care from another homeless person and his partner who shared some of their sparse resources (i.e., wound dressings and disinfectants). He used the digital camera provided as part of the study to keep track of the extent of the inflammation. Nonetheless, Nate had to return to the Emergency Department for an antibiotics script almost a week after his first attempt to present to a mainstream GP. In the public health record, Nate could possibly be seen as misutilising acute care services. The framing as an issue of ‘inappropriate use’ owed‐to the health‐seeking behaviours of the person with lived experience of homelessness obscures the socially embedded nature of relations of care. The experience of being turned away, not only resulted in the exacerbation of Nate's immediate problems (i.e., wound inflammation, capacity to generate income and sustain accommodation) but eventually led to sepsis, surgery, hospitalization and eroded a longer‐term disposition to engage with primary health care. Primary health care models such as the inclusive health centre where this research took place exist to provide an alternative for people like Nate to reduce such occurrences.

## 4 DISCUSSION

We explored how people with lived experience of homelessness are made to feel welcome, seen, and safe in an inclusive primary health care setting. A better understanding of the structural conditions for service delivery, accessibility to and engagement with these services can promote effective caring relations. This study's findings indicate that spatial and temporal configurations are important but must be interpreted within their capacity to affect relations of care.

Rigid approaches to delivering health care, which is at a certain time, for a determined duration and at a designated location can undermine the reciprocal relationships between services users and providers that promote feelings of welcomeness. As has been found elsewhere, health services catering to people who experience homelessness must acknowledge the agency people are used to exercise in their lives in which the absence of support networks has fostered an intense sense of self‐reliance.^25^ Our findings further strengthen conclusions from previous studies highlighting the important contributions of non‐clinical staff to care work. As Solimeo and colleagues noted,^33^ ^,p.103^ ‘clerks are not simply gatekeepers minding the boundaries between illness and disease’. It is crucial to acknowledge the necessity for skilled staff—both clinical and non‐clinical ‐ aligned with core service values to sustain a relational model of care. We found non‐clinical staff enabled safe and welcoming environments in which people seeking care were seen. The notion of ‘being seen’ by a service provider is multilayered; it captures the capacity to seek advice and treatment as permitted within practice hours, that is timely and affordable. It also means to feel understood within the complexity of one's lived experience free of judgment and with a degree of ownership over how care is done. This study's findings reinforce calls to prioritize relationship building to achieve better health outcomes. It is not the built environment on its own that does the heavy lifting in making service users feel welcome, but the way in which this is drawn on to support caring relations in which everyone can feel valued.

This relational model also aligns with trauma‐informed approaches that allow for service user needs to guide consultations in terms of time needed, in turn facilitating feelings of safety.^32^ Appointment duration has been identified as an important factor in delivering inclusive health care elsewhere,^4^, ^13^, ^22^, ^23^ emphasizing the need to allow for sufficient time to address the complexity of health and social support people with lived experience of homelessness often require. Longer appointment times provide greater opportunity for explanations of disease, are important for educating about risk factors, disease prevention, diagnostic procedures, and the spectrum of available treatments, serving to improve patient health literacy and minimise confusion.^4^ Fewer time constraints can also enhance cultural safety. For instance, Indigenous Australians voice a desire for engagement with health systems that are culturally sensitive and that allow time for considered conversation and connection encapsulated in the concept of ‘yarning’.^34^ Success of yarning programs in healthcare settings for Indigenous Australians indicates that more relaxed interactions which allow for story sharing are preferred over structured appointments with time limits.^35^

The findings presented here add nuance to this insight, by showing that not necessarily the standardization of bookings along longer time slots makes service users feel seen, but the flexibility to afford patients as much or as little time as they need. We note that initial appointments in which the foundations for relational investment are laid, complex medical histories are shared, privacy and administrative processes are navigated require more extensive time commitments. Once a service provider‐service user relationship is established, long appointments are not always desirable, if matched by flexibility in attendance policies. The structure that booked appointments offer may be appreciated by some as a domain of predictability in otherwise chaotic lives. At the Centre where this study took place, a mix between drop‐in and appointment modalities were practiced. While the GP clinic operated on an appointment‐only system, administrative staff went to great lengths to remind patients of their upcoming consultations, assist with scheduling follow‐up, arrange free transport through a full‐time driver employed by the Centre, pivot to telehealth ad hoc as required, and complement the standard system with casual bookings to accommodate urgent presentations. The latter also served to fill slots freed up by last minute cancellations and non‐attendances. In turn, the wellness space de facto operated on a drop‐in basis during the study period, but also noted appointment times for patients who requested them. These flexible service structures were appreciated by service users with lived experience of homelessness.

The present study is not without limitations. While many study participants touched upon the relevance of remote health care delivery relying on digital information and communication technology, it is beyond scope to canvass the advantages and disadvantages of telehealth for people experiencing homelessness. As a means to check in or avoid that a consultation is missed, telehealth offers opportunities to manage ongoing health concerns remotely with brief time commitment, and to nurture relationships in which service users feel cared for and about. We believe that this area merits more profound exploration, given that telehealth has seen a boost in practice implementation and is likely to become a permanent feature of the primary health care landscape. Our findings are based on extensive qualitative data from multiple stakeholders using interview, observation, and visual modalities. As for all qualitative work, these findings need to be situated within their context. In this way, the lessons learned from the Centre at which this study was conducted provide important insights into underlying service principles for those who wish to leverage the potential of appropriate spatio‐temporal configurations to support the relational delivery of health care.

## 5 CONCLUSION

It is well‐documented how meeting the health needs of people experiencing homelessness through primary health care reduces burden on health systems.^13^, ^36^ Beyond the logic of cost and benefit for the wider community, greater accessibility of primary health care eases the considerable human suffering that comes with the exacerbation of acute and chronic conditions people with experience of homelessness disproportionally face. This study shows that people *do* attempt to seek primary care in a setting where they are made to feel welcome, safe, and seen. Spatio‐temporal configurations of primary care that allow for flexibility can support a relational model of care in which vulnerabilities can be acknowledged, shared, and addressed. Temporal affordances for appointment time and duration as well as a suitable mix of in‐person, outreach and in‐reach modalities paired with non‐punitive attendance management combine to promote relational investment. Yet, due to the socially embedded nature of health care, concerns of financial viability sit uneasily alongside commitments to social justice. We conclude that structural change that facilitates financial viability within the larger funding system in which primary health care is provided could result in greater cost savings for the public purse, better experiences with health services utilisation for people who experience homelessness and ultimately better health outcomes.

## AUTHOR CONTRIBUTIONS

Stefanie Plage has conceived of the project and study design, undertook data collection, analysis and reporting, Kirsten Baker contributed to data collection, literature review, analysis and reporting, Cameron Parsell contributed to data collection, analysis and reporting. Rose‐Marie Stambe contributed to data collection, analysis and reporting. Ella Kuskoff contributed to data collection, analysis and reporting, Arif Mansuri contributed to data collection and reporting. All authors have read and approved the final manuscript.

## CONFLICT OF INTEREST STATEMENT

The authors declare no conflict of interest.

## Data Availability

All data on which this analysis is based are stored and accessible at UQ's research data management (RDM) server. Reasonable requests for the data that support the findings of this study will be considered subject to ethical review and approval of UQ's Human Research Ethics Committee (HREC). Such requests should be directed to the corresponding author. The data are not publicly available due to privacy or ethical restrictions.
